# Kindlin-2 Promotes Chondrogenesis and Ameliorates IL-1beta-Induced Inflammation in Chondrocytes Cocultured with BMSCs in the Direct Contact Coculture System

**DOI:** 10.1155/2022/3156245

**Published:** 2022-04-12

**Authors:** Zhefeng Chen, Kai Shen, Ziyang Zheng, Jinchun Zhou, Shujie Zhao, Huanghe Song, Jiuxiang Liu, Xuan Zhao, Feng Liu, Qiang Zuo

**Affiliations:** Department of Orthopedics, The First Affiliated Hospital of Nanjing Medical University, Nanjing 210029, China

## Abstract

The osteoarthritis caused by trauma or inflammation is associated with severe patient morbidity and economic burden. Accumulating studies are focusing on the repair of articular cartilage defects by constructing tissue-engineered cartilage. Recent evidence suggests that optimizing the source and quality of seed cells is one of the key points of cartilage tissue engineering. In this study, we demonstrated that Kindlin-2 and its activated PI3K/AKT signaling played an essential role in promoting extracellular matrix (ECM) secretion and ameliorating IL-1beta-induced inflammation in chondrocytes cocultured with bone marrow stem cells (BMSCs). *In vivo* experiments revealed that coculture significantly promoted hyaline cartilage regeneration. *In vitro* studies further uncovered that chondrocytes cocultured with BMSCs in the direct contact coculture system upregulated Kindlin-2 expression and subsequently activated the PI3K/AKT signaling pathway, which not only increases Sox9 and Col2 expression but also restores mitochondrial membrane potential and reduces ROS levels and apoptosis under inflammatory conditions. Overall, our findings indicated that direct contact BMSC-chondrocyte coculture system could promote chondrogenesis, and identified Kindlin-2 represents a key regulator in this process.

## 1. Introduction

Articular cartilage damaged by trauma or inflammatory factors is often difficult to repair effectively and eventually develops into osteoarthritis (OA) [1, 2]. The incidence of OA is increasing yearly and has become a serious disease threatening human health. Investigations have indicated that the incidence of OA in the United States is as high as 50% [3, 4]. In the middle-aged and elderly in China, lumbar osteoarthritis was the most prevalent with a prevalence of 25.03%, followed by the prevalence of knee osteoarthritis, which was 21.51% [5]. However, despite its prevalence and severity, there is still no curative or effective treatment due to our limited understanding of the pathogenesis of osteoarthritis. The repair of articular cartilage defects by constructing tissue-engineered cartilage has been a hot area of research in recent years, among which how to further optimize the source and quality of seed cells is still one of the key and difficult points of cartilage tissue engineering.

Chondrocytes and BMSCs are commonly used seed cells at present. Both of them have defects in the construction of tissue-engineered cartilage [6]. Primary chondrocytes are relatively limited in source and tend to lose their special phenotypes after being passaged several times *in vitro*. Exogenous cytokines must be provided for BMSCs to maintain chondrogenic differentiation. Researchers tried to coculture the two cell lines, and the results showed that coculture could increase the synthesis of chondrogenic ECM components such as type II collagen (Col2) and aggrecan (Aggrecan) [7, 8].

Kindlin-2 belongs to a family of conserved cytoplasmic proteins. Kindlin-2 promotes cell differentiation, survival, and migration by interacting with Integrin [9] and is involved in mediating integrin's regulation of cell propagation [10] and intercellular communication [11]. Deficiency of Kindlin-2 expression can lead to abnormal chondrogenesis and affect the survival of chondrocytes. Global inactivation of Kindlin-2 in mice resulted in early embryonic lethality at E7.5 [12].

To explore the role of Kindlin-2 in promoting specific ECM synthesis in the direct contact coculture system, we specifically knocked down and overexpressed Kindlin-2 in chondrocytes and constructed a direct contact coculture system of chondrocytes and BMSCs. We found that Kindlin-2 can promote the function of chondrocytes, regulate the synthesis and secretion of ECM of chondrocytes, and affect the activation of the PI3K/AKT signaling pathway in the direct contact coculture system. In addition, we also demonstrated the protective effect of Kindlin-2 and its activated PI3K/AKT signaling pathway on chondrocytes in an inflammatory environment, specifically by restoring mitochondrial membrane potential and reducing ROS levels and apoptosis. These findings identified Kindlin-2-overexpressing chondrocytes as potential seed cells to construct high-quality tissue-engineered cartilage.

## 2. Results

### 2.1. Kindlin-2 Promotes Chondrogenesis in the Direct Contact Coculture System

**Figure 1:**
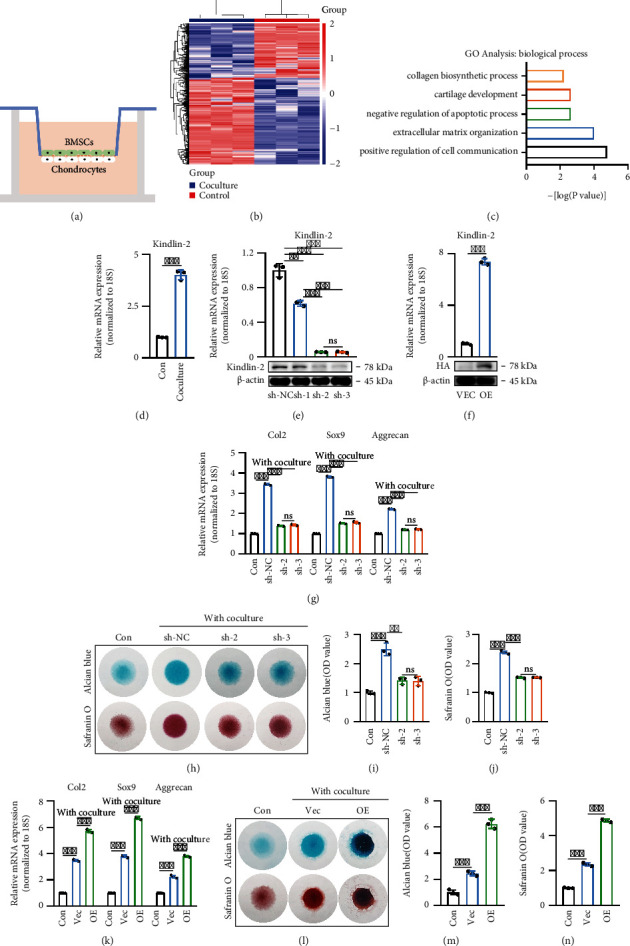
Kindlin-2 promotes chondrogenesis in the direct contact coculture system. (a) A direct contact BMSC-chondrocyte coculture system to evaluate the effects of BMSCs on chondrocytes. (b) Heatmap of DEGs of chondrocytes cocultured with or without BMSCs. Green and red colors represent low and high expression values, respectively. (c) Representative biological process (BP) categories identified in GO analyses based on upregulated DEGs in cocultured chondrocytes compared with control counterparts. (d) Expression pattern of Kindlin-2 in chondrocytes cocultured with or without BMSCs were determined using RT–qPCR. Values are expressed as mean ± s.d., ^∗∗∗^*p* < 0.001. (e) The knockdown efficiency of Kindlin-2 in chondrocytes was confirmed by qPCR and Western blotting. Values are expressed as mean ± s.d., ^∗∗^*p* < 0.01, ^∗∗∗^*p* < 0.001, ns indicates no significance. (f) Overexpression efficiency of Kindlin-2 in chondrocytes was confirmed by qPCR and Western blotting. Values are expressed as mean ± s.d., ^∗∗∗^*p* < 0.001. (g) mRNA expression levels of cartilage-specific genes (Col2, Sox-9, and Aggrecan) in chondrocytes on day 7 were detected by qPCR in different groups. 18S was used as an internal control. Values are expressed as mean ± s.d., ^∗∗^*p* < 0.01, ns indicates no significance. (h) In a direct contact coculture system for 7 days, knockdown of Kindlin-2 attenuated the regulation of BMSCs on chondrocyte ECM secretion, as indicated by Alcian blue staining and safranin O staining. Chondrocytes without coculture were used as the control (Con) group. (i, j) Quantitative evaluation of Alcian blue staining results (i) and safranin O staining results (j) on day 7. Values are expressed as mean ± s.d., ^∗∗^*p* < 0.01, ^∗∗∗^*p* < 0.001, ns indicates no significance. (k) mRNA expression levels of Col2, Sox-9, and Aggrecan in chondrocytes on day 7 by qPCR in different groups. 18 s was used as an internal control. Values are expressed as mean ± s.d., ^∗∗∗^*p* < 0.001, ns indicates no significance. (l) In the direct contact coculture system, Kindlin-2-overexpressing chondrocytes enhanced chondrogenic ability on day 7, as revealed by Alcian staining and safranin O staining. Chondrocytes cultured alone were set as the Con group. Vec: vector group; OE: overexpression group. (m, n) Quantitative analyses of Alcian staining results (m) and safranin O staining results (n) on day 7 were performed. Values are expressed as mean ± s.d., ^∗∗∗^*p* < 0.001.

Chondrocytes cocultured with BMSCs can increase the synthesis of specific ECM components, such as Col2, Sox9, and aggrecan. BMSCs can provide nutritional support to chondrocytes in the direct contact coculture system [13, 14]. To explore the key regulator of chondrogenesis in the direct contact coculture system (Figure 1(a)), we performed transcriptome analysis by high-throughput RNA sequencing (RNA-Seq) using 3 biological replicates of chondrocytes cocultured with or without BMSCs in a direct contact coculture system (Figure 1(b)). The expression level of Kindlin-2 was upregulated by 5.18 times. Compared with their control counterparts, cocultured chondrocytes exhibited increased cartilage development and extracellular matrix organization (Figure 1(c)). The results of RT–qPCR also confirmed that coculturing with BMSCs significantly upregulated the Kindlin-2 mRNA expression level in chondrocytes (Figure 1(d)). Kindlin-2 was recently reported to be an important factor regulating the proliferation and survival of chondrocytes, and defects in the expression of Kindlin-2 can lead to abnormal chondrogenesis and affect the survival of chondrocytes [15]. Therefore, *in vitro*, we explored whether Kindlin-2 was involved in the regulation of BMSCs on chondrocytes. As shown in Figures 1(e) and 1(f), Kindlin-2 was knocked down and overexpressed on chondrocytes, which were confirmed by qPCR and Western blotting. We chose sh-2 and sh-3 groups for further investigations. As expected, the mRNA expression levels of chondrocyte-specific biomarkers (Col2, Sox9, and Aggrecan) indicated that the knockdown of Kindlin-2 attenuated the regulation of BMSCs on chondrocytes in the direct contact coculture system (Figure 1(g)). These results were confirmed by Alcian blue and safranin O staining and quantitative analysis after chondrocytes were micromass cocultured with BMSCs (Figures 1(h)–1(j)).

The mRNA expression values of Col2, Sox9, and Aggrecan in Kindlin-2-overexpressing chondrocytes showed that Kindlin-2-overexpressing chondrocytes cocultured with BMSCs in the direct contact coculture system could significantly improve the regulation of BMSCs on chondrocytes and promote specific ECM synthesis (Figure 1(k)). Similarly, Kindlin-2-overexpressing chondrocytes were micromass cocultured with BMSCs for 7 days and then stained with Alcian blue and Safranin O (Figures 1(l)–1(n)). Collectively, these results suggest that Kindlin-2 can promote chondrogenesis in the direct contact coculture system.

### 2.2. *In Vivo* Cartilage Regeneration in Nude Mice

**Figure 2:**
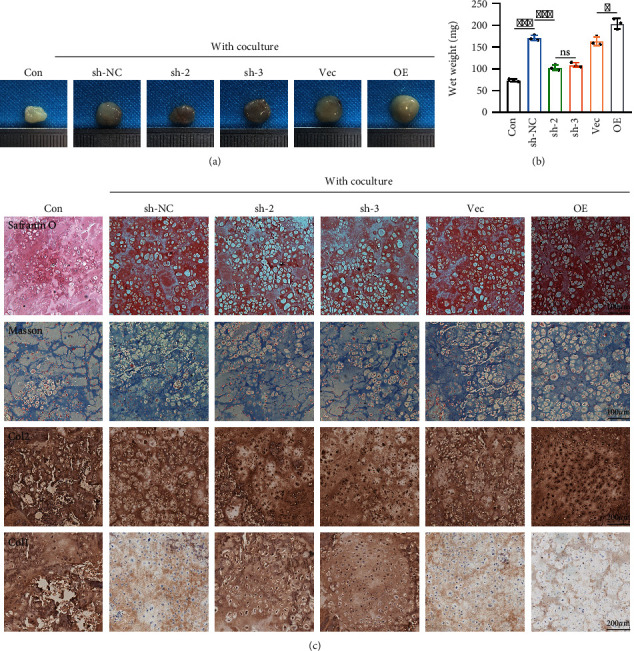
*In vivo* cartilage regeneration in nude mice. (a) Gross morphology examination after hydrogels were injected subcutaneously into nude mice for 4 weeks. (b) Implants containing Kindlin-2 overexpression or knockdown chondrocytes cocultured with BMSCs were analyzed by wet weight. Values are expressed as mean ± s.d., ^∗^*p* < 0.05, ^∗∗∗^*p* < 0.001, ns indicates no significance. (c) Implants containing Kindlin-2 overexpression or knockdown chondrocytes cocultured with BMSCs were analyzed by Safranin O, Masson, collagen I, and collagen II staining confirmed by histological examination and immunohistochemical staining.

To investigate the function of chondrocytes that synthesize and secrete ECM in the direct contact coculture system and their ability to support new cartilage formation *in vivo*, similar volumes of different groups of chondrocytes were subcutaneously injected into nude mice. Implants were removed from the subcutaneous space and analyzed after 4 weeks. As shown in Figure 2(a), implants in the coculture group were larger and more compact than those in the control group, and the kinidlin-2 overexpression coculture group was the largest.

The wet weight results showed that the implants in the coculture group were larger than those in the control group. In the coculture groups, the implants in the Kindlin-2 overexpression coculture group were the largest, followed by the NC group, and the Kindlin-2 knockdown coculture group was the smallest (Figure 2(b)). Therefore, the increase in cartilage regeneration was mediated by Kindlin-2. Histological examination and immunohistochemical staining confirmed that the coculture groups had stronger Safranin O staining, Masson staining, and collagen II staining and weaker collagen I staining. Consistent with the wet weight results, the Kindlin-2-overexpressing coculture group synthesized and secreted the largest amount of ECM *in vivo* (Figure 2(c)). These findings demonstrate that the Kindlin-2 overexpression coculture group significantly promoted hyaline cartilage regeneration and cartilage lacuna formation, which is similar to native cartilage.

### 2.3. Potential Regulatory Role of Chondrocyte Kindlin-2 in PI3K/AKT/mTOR Signaling in the Direct Contact Coculture System

**Figure 3:**
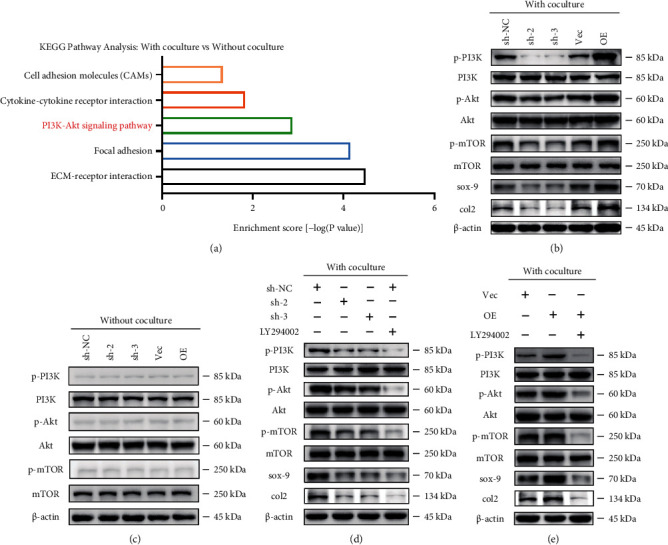
Potential regulatory role of chondrocyte Kindlin-2 in PI3K/AKT/mTOR signaling in the direct contact coculture system. (a) Representative upregulated KEGG pathway categories affected by coculturing with BMSCs. (b) Altered protein expression levels of p-PI3K/PI3K, p-AKT/AKT, p-mTOR/mTOR, Col2, and aggrecan were detected using Western blotting in Kindlin-2 knockdown and overexpression chondrocytes in the direct contact coculture system. (c) Immunoblot images showing the effect of Kindlin-2 knockdown or overexpression on the expression of p-PI3K/PI3K, p-AKT/AKT, and p-mTOR/mTOR in chondrocytes without coculturing. (d) Protein expression levels of p-PI3K/PI3K, p-AKT/AKT, p-mTOR/mTOR, Col2, and aggrecan were detected using Western blotting in Kindlin-2 knockdown and overexpression chondrocytes in the direct contact coculture system or Kindlin-2 sh-NC chondrocytes treated with LY294002 (an inhibitor of PI3K) before coculture. (e) Protein expression levels of p-PI3K/PI3K, p-AKT/AKT, p-mTOR/mTOR, Col2, and aggrecan were detected using Western blotting in Kindlin-2 knockdown and overexpression chondrocytes in the direct contact coculture system or Kindlin-2 overexpression chondrocytes treated with LY294002 (an inhibitor of PI3K) before coculture.

A KEGG pathway analysis of differentially expressed genes between chondrocytes cocultured with BMSCs in the direct contact coculture system and chondrocytes cultured alone was performed to further identify the molecular mechanisms by which Kindlin-2 regulates the synthesis and secretion of ECM of chondrocytes. As shown in Figure 3(a), the PI3K/AKT signaling pathway was markedly activated in chondrocytes cocultured with BMSCs in the direct contact coculture system compared to control chondrocytes. The results revealed that genes related to the PI3K/AKT signaling pathway were significantly enhanced in the coculture group, suggesting a potential regulatory role of Kindlin-2 in the PI3K/AKT signaling pathway. Chondrocytes (the only cell type in cartilage) are the dominant influence on the health and function of cartilage [16], and PI3K/AKT signaling pathway is a vital regulator of chondrocyte survival and apoptosis [17]. As indicated in Figure 3(b), the PI3K/AKT/mTOR signaling pathway was activated, and knockdown of Kindlin-2 decreased, while overexpression of Kindlin-2 increased the levels of phosphorylated PI3K, AKT, and mTOR in the direct contact coculture system. However, none of the phosphorylated PI3K, AKT, and mTOR levels were altered without coculturing (Figure 3(c)). Meanwhile, in coculture system, the results showed that inhibition of Kindlin-2 reduced the activation of PI3K/AKT/mTOR signaling pathway even though the expression of PI3K was upregulated (Supplementary Figure 1A–1B). Furthermore, when the expression of PI3K was inhibited, the activation of PI3K/AKT/mTOR signaling pathway in Kindlin-2 overexpressed group was also inhibited (Supplementary Figure 1C–1D).

To further confirm that the PI3K/AKT/mTOR signaling pathway is involved in Kindlin-2-mediated ECM secretion of chondrocytes, a small molecule inhibitor targeting PI3K (LY294002) was used. As shown in Figure 3(d), PI3K/AKT/mTOR signaling pathway was inhibited by LY294002 in chondrocytes cocultured with BMSCs in the direct contact coculture system. As expected, the expression levels of Col2 and Sox9 decreased after treatment with LY294002, as indicated by the weaker band seen in Figure 3(d). Similar results were observed when Kindlin-2-overexpressing chondrocytes were treated with LY294002 before coculturing (Figure 3(e)). These results were consistent with our RNA sequencing data, which suggested that Kindlin-2 could be a major regulator of the PI3K/AKT signaling pathway in the direct contact coculture system.

### 2.4. Kindlin-2-Mediated PI3K/AKT Signaling Pathway in Chondrocytes Is Essential for Chondrogenesis

**Figure 4:**
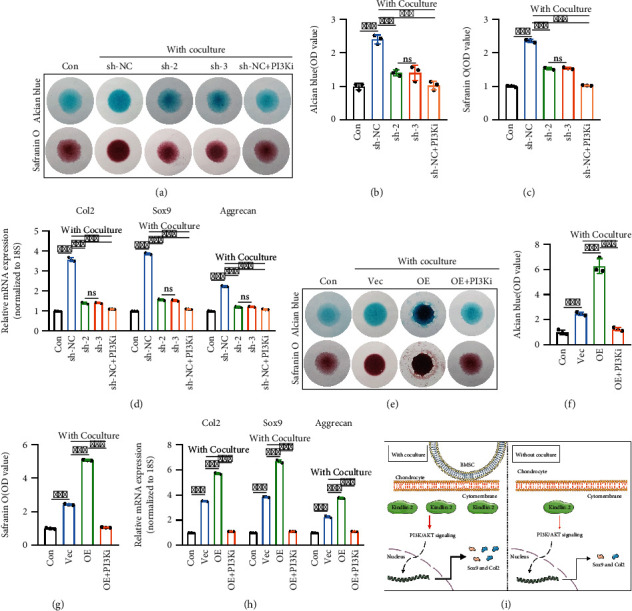
Kindlin-2-mediated PI3K/AKT/mTOR signaling pathway in chondrocytes is essential for chondrogenesis. (a–c) In the direct contact coculture system, knockdown of Kindlin-2 or inhibition of PI3K/AKT/mTOR signaling in chondrocytes reduced cartilage matrix formation, as observed by Alcian blue staining and safranin O staining (a). Quantitative evaluation of Alcian blue staining results (b) and safranin O staining results (c) on day 7 was performed. Chondrocytes without coculture were used as the Con group. Values are expressed as mean ± s.d., ^∗∗∗^*p* < 0.001, ns indicates no significance. (d) mRNA expression levels of cartilage-specific genes (Col2, Sox-9, and Aggrecan) in chondrocytes on day 7 were detected by qPCR in different groups. 18S was used as an internal control. Values are expressed as mean ± s.d., ^∗∗∗^*p* < 0.001, ns indicates no significance. (e–g) Inhibition of PI3K/AKT/mTOR signaling in Kindlin-2 OE chondrocytes in the direct contact coculture system decreased cartilage matrix formation, as observed by Alcian blue staining and safranin O staining (e). Quantitative evaluation of Alcian blue staining results (f) and safranin O staining results (g) on day 7 was performed. Values are expressed as mean ± s.d., ^∗∗∗^*p* < 0.001. (h) mRNA expression levels of cartilage-specific genes (Col2, Sox-9, and Aggrecan) in chondrocytes on day 7 were detected by qPCR in different groups. 18S was used as an internal control. Values are expressed as mean ± s.d., ^∗∗∗^*p* < 0.001. (i) Schematic of the functional consequences and specific mechanism of chondrocytes cocultured with BMSCs in the direct contact coculture system.

We further confirmed that Kindlin-2-mediated synthesis and secretion of chondrocyte ECM in the direct contact coculture system were regulated by activation of the PI3K/AKT signaling pathway. Chondrocytes and BMSCs were cocultured in micromass, as shown in Figures 4(a)–4(c). Alcian blue and safranin O staining and subsequent quantitative analysis showed that the synthesis and secretion of chondrocyte ECM could be significantly inhibited by the small molecule inhibitor LY294002 in the direct contact coculture system. These results were also confirmed by the mRNA expression levels of Col2, Sox9, and Aggrecan (Figure 4(d)).

In addition, analysis of Alcian blue, safranin O staining, and mRNA expression levels of Kindlin-2-overexpressing chondrocytes cocultured with BMSCs after the use of LY294002 confirmed that the ECM secretion promoting effect of Kindlin-2 in chondrocytes was modulated by PI3K/AKT signaling pathway (Figures 4(e)–4(h)), which is consistent with previous results.

As shown in Figure 4(i), chondrocytes were cocultured with BMSCs in a direct contact way, and Kindlin-2 expression was upregulated. Subsequently, activation of the PI3K/AKT signaling pathway was shown to increase Sox9 and Col2 expression, which facilitates the synthesis and secretion of ECM of chondrocytes. Overall, our findings provide a novel mechanistic basis for the interplay between chondrocytes and BMSCs in a direct contact coculture system.

### 2.5. Kindlin-2-Mediated PI3K/AKT Pathway Protects Chondrocytes against IL­1beta-Induced Inflammation

**Figure 5:**
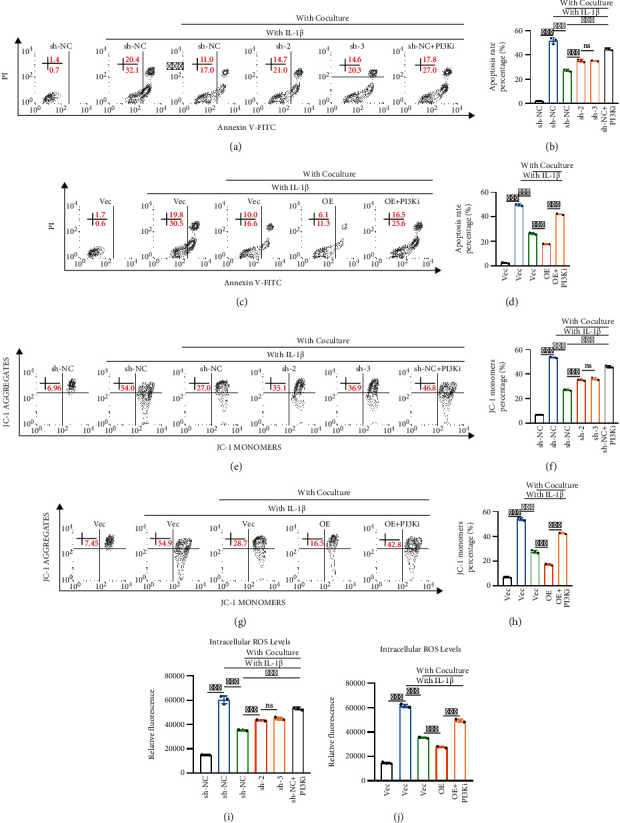
Kindlin-2-mediated PI3K/AKT pathway protects chondrocytes against IL­1beta-induced inflammation. (a, b) Knockdown of Kindlin-2 or inhibition of PI3K/AKT/mTOR signaling in chondrocytes increased IL-1beta-induced chondrocyte apoptosis, as detected by Annexin V-FITC/PI staining and flow cytometry. Quantitative evaluation of Annexin V-FITC/PI staining results was performed. Values are expressed as mean ± s.d., ^∗∗∗^*p* < 0.001, ns indicates no significance. (c, d) Inhibition of PI3K/AKT/mTOR signaling in Kindlin-2 OE chondrocytes in the direct contact coculture system increased IL-1beta-induced chondrocyte apoptosis, as detected by Annexin V-FITC/PI staining and flow cytometry. Quantitative evaluation of Annexin V-FITC/PI staining results was performed. Values are expressed as mean ± s.d., ^∗∗∗^*p* < 0.001. (e, f) Knockdown of Kindlin-2 or inhibition of PI3K/AKT/mTOR signaling in chondrocytes further reduced mitochondrial membrane potential induced by IL-1beta, as detected by JC-1 dye and flow cytometry. Quantitative evaluation of the percentage of collapsed mitochondrial membrane potential is expressed as mean ± s.d., ^∗∗∗^*p* < 0.001, ns indicates no significance. (g, h) Inhibition of PI3K/AKT/mTOR signaling in Kindlin-2 OE chondrocytes in the direct contact coculture system reduced the mitochondrial membrane potential induced by IL-1beta, as detected by JC-1 dye and flow cytometry. Quantitative evaluation of the percentage of collapsed mitochondrial membrane potential is expressed as mean ± s.d., ^∗∗∗^*p* < 0.001. (i) Knockdown of Kindlin-2 or inhibition of PI3K/AKT/mTOR signaling in chondrocytes increased ROS levels induced by IL-1beta, as determined by DCHDA assay. Values are expressed as mean ± s.d., ^∗∗∗^*p* < 0.001, ns indicates no significance. (j) Inhibition of PI3K/AKT/mTOR signaling in Kindlin-2 OE chondrocytes in the direct contact coculture system increased ROS levels induced by IL-1beta, as determined by DCHDA assay. Values are expressed as mean ± s.d., ^∗∗∗^*p* < 0.001.

Among the proinflammatory cytokines involved in OA, IL­1beta is considered one of the major players; IL­1beta seems to be associated with cartilage destruction [18]. To simulate the proinflammatory and catabolic effects of IL­1beta on chondrocytes during the process of osteoarthritis *in vitro*, chondrocytes were treated with IL-1beta with or without direct contact coculture for 48 h. The results showed that the percentage of apoptotic chondrocytes (early and late apoptosis) in the direct contact coculture system was significantly decreased compared with that of control chondrocytes after treatment with IL-1beta for 48 h. Knockdown of Kindlin-2 attenuated the protective effect of direct contact coculture on chondrocytes through the PI3K/AKT pathway, which was further confirmed by the addition of the small molecule inhibitor LY294002 (Figures 5(a) and 5(b)). Similar results were observed when Kindlin-2-overexpressing chondrocytes were cocultured with BMSCs in the direct contact coculture system, and LY294002 was used (Figures 5(c) and 5(d)).

The mitochondrial membrane potential is essential for maintaining mitochondrial oxidative phosphorylation and ATP production. The decrease in mitochondrial membrane potential is one of the symbolic events of apoptosis. As Figures 5(e)–5(h) shows, there was a significant decrease in mitochondrial membrane potential after chondrocytes were treated with IL-1beta for 48 h. Direct contact coculture restored the mitochondrial membrane potential, whereas the small molecule inhibitor LY294002 abolished this effect. Knockdown of Kindlin-2 attenuated, while overexpression of Kindlin-2 enhanced, the restoration of mitochondrial membrane potential.

There is a substantial body of published research that suggests ROS are a major causative factor for OA development. Oxidative stress elicited by ROS is capable of oxidizing and subsequently disrupting cartilage homeostasis and promoting catabolism via induction of cell death [19]. Using the DCFDA fluorescent probe, we showed that IL-1beta significantly increased intracellular ROS production, while chondrocytes cocultured with BMSCs decreased ROS production. Chondrocytes regulate ROS production in the direct contact coculture system in a Kindlin-2-mediated PI3K/AKT manner, as indicated in Figures 5(i) and 5(j).

## 3. Discussion

Kindlin-2 has been reported to play important roles in fibrosis and cancer [20, 21]. Additionally, published data suggested that Kindlin-2 protects chondrocytes from apoptosis and serves as a major controlling factor in the regulation of the chondrocyte differentiation program and chondrogenesis during prenatal and postnatal skeletal development [15]. In the present study, we demonstrated that Kindlin-2-mediated PI3K/AKT signaling not only promotes the synthesis and secretion of chondrocyte ECM but also regulates the inflammatory responses of chondrocytes in the direct contact coculture system.

Accumulating evidence has indicated that the crosstalk between BMSCs and chondrocytes has a great impact on cartilage matrix formation and may have therapeutic potential for cartilage regeneration [22]. However, the underlying mechanisms of this communication between BMSCs and chondrocytes need to be further characterized. BMSC-derived exosomes can effectively promote cartilage repair and extracellular matrix synthesis [23]. In addition, FGF-1, VEGF-A, and PDGFbb secreted by BMSCs exert modulatory effects on chondrocytes, including changes in cell shape, proliferation, gene expression, and ECM production [24]. Our study shows that Kindlin-2 contributes to increased cartilage formation in a direct contact BMSC-chondrocyte coculture system, which provides a new idea to explain the better performance of chondrocytes in the direct contact coculture system.

A previous study determined that Kindlin-2 played a critical role in the regulation of TGF-*β* signaling during the chondrogenic differentiation program. Additionally, Kindlin-2 regulated chondrocyte function and chondrogenesis by controlling the expression of Sox9, a master regulator of chondrocyte function [15]. Our study first uncovered that chondrocyte Kindlin-2-mediated PI3K/AKT signaling pathway is the key regulator of ECM secretion, apoptosis, mitochondrial membrane potential, and ROS production of chondrocytes in the direct contact coculture system, providing an explanation as to why Kindlin-2-overexpressing chondrocytes are suitable as seed cells for tissue engineering. In addition, the regulatory effect and mechanism of Kindlin-2 on chondrocytes are worthy of further exploration.

Various molecules, including glucose, insulin, and many growth factors and cytokines can initiate PI3K/AKT signaling [25]. Numerous evidences supported the involvement of PI3K/AKT signaling pathway in chondrocyte survival and apoptosis. 17*β*-estradiol- (E2-) mediated PI3K/AKT activation significantly promoted chondrocyte proliferation in a rat OA model [26]. Multiple growth factors, such as FGF1827, IGF-1, and platelet-derived growth factors, could rescue IL-1beta-induced increase in mitochondrial-related apoptosis by the activation of PI3K/AKT [27, 28]. Collectively, similar to previous studies, our results also proved the important role of PI3K/AKT signaling pathway in chondrocyte proliferation and apoptosis.

In conclusion, we provide new insights into the regulatory effect and specific mechanism of Kindlin-2 in chondrocytes cocultured with BMSCs in the direct contact coculture system. Kindlin-2 is essential for regulating chondrocyte function and ECM secretion during chondrogenesis. Furthermore, our study suggests that Kindlin-2-mediated PI3K/AKT signaling pathway controls apoptosis, mitochondrial membrane potential, and ROS production in response to IL-1beta. Our work may provide the possibility of constructing high-quality tissue-engineered cartilage with Kindlin-2-overexpressing chondrocytes instead of conventional seed cells.

## 4. Materials and Methods

### 4.1. Cell Culture and Reagents

Primary chondrocytes were obtained from the femoral condyles and tibial plateau of 5-day-old C57BL/6 mice. In brief, after washing with PBS, the cartilage was cut into pieces. Next, cartilage chips were sequentially incubated with trypsin–EDTA solution and 3 mg/mL collagenase II (C6885, Sigma–Aldrich) for 3-4 h at 37°C, and the digests were filtered through a 70 *μ*m cell strainer. Cells were plated in DMEM/F12 (11320033, Thermo Fisher Scientific, USA) supplemented with 10% fetal bovine serum (FBS, 10099141, Thermo Fisher Scientific, USA) and 1% penicillin/streptomycin (10378016, Thermo Fisher Scientific, USA). Primary BMSCs were obtained and cultured as previously described [29, 30]. Chondrocytes were cocultured with BMSCs at a 3 : 1 ratio in a direct contact BMSC-chondrocyte coculture system as reported previously [31]. Cells were cultured in DMEM/F12 (11320033, Thermo Fisher Scientific, USA) containing 10% fetal bovine serum (FBS) and 1% penicillin/streptomycin. The medium was changed every two days. After incubation for 7 days, chondrocytes were digested for further evaluations.

### 4.2. Animal Models

Cells were harvested and suspended at a concentration of 5 × 10^6^ cells/mL in silk fibroin (SF) solution, which was prepared from *Bombyx mori silkworm cocoons* at concentrations of 4% (*w*/*v*) as described in previous studies [32]. For the coculture groups, chondrocytes and BMSCs were mixed at a 3 : 1 ratio. Next, the solution was divided into 200 *μ*L aliquots, and hydrogels were subcutaneously injected into nude mice three days after gelation. Finally, implants were removed from the subcutaneous space and for further analysis after 4 weeks. All animal experimental procedures were approved by the Nanjing Medical University Animal Ethical Committee (NJMU/IACUC 2007029, 2103044). Furthermore, all animal procedures were performed in accordance with the guidelines for the care and use of laboratory animals of Nanjing Medical University.

### 4.3. RNA Isolation and qPCR

Total RNA of cells was extracted by using TRIzol reagent (Takara, Japan), and HiScript II Q RT SuperMix for qPCR (R122-01, Vazyme, Nanjing, China) was used to transcribe RNA into cDNA following the manufacturer's instructions. Next, quantitative real-time qPCR was analyzed using AceQ qPCR SYBR Green Master Mix (Q111-02, Vazyme, China) in a 7500 real-time PCR system (Applied Biosystems, Inc., USA). The primer sets used were Kindlin-2: sense TGGACGGGATAAGGATGCCA, anti-sense TGACATCGAGTTTTTCCACCAAC; Col2: sense CCACACCAAATTCCTGTTCA, anti-sense ACTGGTAAGTGGGGCAAGAC; Sox9: sense CCACGGAACAGACTCACATCTCTC, anti-sense CTGCTCAGTTCACCGATGTCCACG; Aggrecan: sense AGGACCTGGTAGTGCGAGTG, anti-sense GCGTGTGGCGAAGAA; 18S: sense CGGCTACCACATCCAAGGAA, anti-sense GCTGGAATTACCGCGGCT. All data were normalized to 18S expression, and relative mRNA expression was assessed.

### 4.4. Western Blotting (WB)

Total protein was extracted from chondrocytes using protein extraction buffer (Beyotime, Shanghai, China). Furthermore, equal amounts of proteins were separated by SDS–PAGE and transferred to a polyvinylidene fluoride (PVDF) membrane. After blocking with 5% skimmed milk, the membrane was probed with the following primary antibodies: anti-*β*-actin (1 : 2000), anti-PI3K (1 : 1000), anti-p-PI3K (1 : 1000), anti-AKT (1 : 1000), anti-p-AKT (1 : 1000), anti-mTOR (1 : 1000), anti-p-mTOR (1 : 1000), anti-Col2 (1 : 1000), anti-Sox-9 (1 : 1000), and species-specific secondary antibodies (1 : 10000). The bands were visualized by the Odyssey imaging system (LI-COR, Lincoln, NE, USA).

### 4.5. Micromass Culture

Primary chondrocytes and BMSCs were digested when they were 80%–90% confluent and resuspended at 1 × 10^7^ cells per milliliter. For the coculture groups, chondrocytes and BMSCs were mixed at a 3 : 1 ratio. Then, the cells were plated in a 12.5 *μ*L droplet of cell suspension in the center of a 12-well plate. After the plate was placed at 37°C for 2 h, DMEM/F12 containing 10% fetal bovine serum (FBS) and 1% penicillin/streptomycin was gently added. On day 7, the micromasses were fixed with 4% paraformaldehyde and used for further analysis.

### 4.6. Alcian Blue Staining, Safranin O Staining, and Masson Staining

Chondrocytes cocultured with or without BMSCs were first fixed with 4% paraformaldehyde for 30-45 min and then stained with Alcian blue (G1027, Servicebio, Wuhan, China) and safranin O (G1053, Servicebio, Wuhan, China) to detect the extent of matrix mineralization. Quantification of Alcian blue staining was performed by measuring the absorbance at 620 nm after dissolving the stained micromass with 6 M guanidine hydrochloride solution [33]. Safranin O staining was washed out in isopropanol and incubated for 30 min with gentle agitation. Each sample was quantified as optical density in a microplate reader at 540 nm [34]. For tissue Masson staining and safranin O staining, the fixed samples were embedded for sectioning and staining.

### 4.7. Annexin V-FITC/PI Staining

An Annexin V Apoptosis Detection Kit (556547, BD, USA) was used to observe the apoptosis rates following the manufacturer's instructions. Briefly, cells were washed with cold PBS twice after the above treatments and harvested in 1X binding buffer at a concentration of 1 × 10^6^ cells/mL. Then, 100 *μ*L of the solution was transferred to a culture tube, and 5 *μ*L of FITC Annexin V and 5 *μ*L PI were added. The tubes were gently vortexed and incubated at room temperature in the dark for 15 min. Finally, 400 *μ*L of 1X binding buffer was added to each tube. Data were acquired by flow cytometry (FACSCalibur, BD) and analyzed using FlowJo software (Version 7.6.1, Treestar, USA).

### 4.8. Mitochondrial Membrane Potential Detection

The mitochondrial membrane potential was detected by a mitochondrial membrane potential assay kit with JC-1 (HY-15534, MCE, USA) following the manufacturer's instructions. Cells from each group were collected into centrifuge tubes, and JC-1 was added to a final concentration of 2 *μ*M. After being incubated at 37°C in the dark for 15-20 minutes, cells were washed with PBS twice and resuspended in 500 *μ*L PBS. Samples were analyzed on a flow cytometer (FACSCalibur, BD).

### 4.9. Measurement of Reactive Oxygen Species

ROS production was tested by using a Reactive Oxygen Species Assay Kit (S0033S, Beyotime, China) following the manufacturer's instructions. Chondrocytes in the indicated groups were collected and resuspended in diluted DCFH-DA probes. Next, the cells were incubated at 37°C in the dark for 20 minutes and washed three times with serum-free culture medium. Finally, ROS production was measured by using a microplate reader at Ex/Em 488/525.

### 4.10. RNA-Seq and Bioinformatics Analysis

Total RNA of chondrocytes cocultured with or without BMSC groups in the direct contact coculture system was extracted and converted into cDNA libraries according to previously reported methods [35], and the libraries were sequenced on the Illumina HiSeq X Ten according to the manufacturer's protocols. The reads were aligned with the TopHat program (version 2.0.11). Additionally, the FPKM values of genes were calculated, Pearson's correlation analysis was performed, and heatmaps were generated. The RNA-seq results were uploaded to the Gene Expression Omnibus (GEO) database with accession number GSE191024. In this study, differentially expressed genes (DEGs) were defined as fold changes > 1.5 and *p* < 0.05. Kyoto Encyclopedia of Genes and Genomes (KEGG) analyses and GO analyses were further performed to interpret the biological significance of DEGs.

### 4.11. Plasmid Construction and Transfection

All plasmids (Kindlin-2, sh-NC and vector) were constructed from GenePharma (Shanghai, China). Virus packaging was performed as previously described [36–38], and titers were also tested. The cells were infected with 1 × 10^8^ lentivirus-transducing units in the presence of 5 *μ*g/mL polybrene (GenePharma, Shanghai, China). After 72 h of culture, infected cells were further selected with 2.5 *μ*g/mL puromycin. The overexpression and knockdown efficacy of Kindlin-2 was verified by qPCR and Western blotting.

### 4.12. Immunohistochemical Staining

The IHC staining was performed as previously reported [37, 38]. Briefly, the slides were blocked by incubation in 10% bovine serum albumin (BSA) followed by incubation with primary antibodies against Col1 (1 : 150) and Col2 (1 : 150). Next, the slides were washed and incubated with the corresponding HPR-conjugated secondary antibody (1 : 300). Furthermore, the proteins were marked with DAB (8059, Cell Signaling Technology, USA) and counterstained with hematoxylin. Finally, images were taken using a microscope (Axio Lab. A1, Zeiss, Heidenheim, Germany).

### 4.13. Statistical Analysis

Quantitative data are presented as the mean ± s.d. and contain at least three independent biological replicates. Unpaired two-tailed Student's *t* test was used for two-group comparisons, and one-way analysis of variance was performed for multigroup comparisons. Differences between groups were considered significant at a *p* value < 0.05.

## Data Availability

The RNA-seq results were uploaded to the Gene Expression Omnibus (GEO) database with accession number GSE191024.

## Supplementary Materials

Supplementary Materials Supplementary Figure 1: Kindlin-2 mediates PI3K/AKT/mTOR signaling pathway in chondrocytes. (A) Overexpression efficiency of PI3K in chondrocytes was confirmed by qPCR and Western blotting. Values are expressed as mean ± s.d., ^∗∗∗^*p* < 0.001. (b) Immunoblot images showing the effect of Kindlin-2 knockdown on the expression of p-PI3K/PI3K, p-AKT/AKT, and p-mTOR/mTOR in PI3K overexpressed chondrocytes in the direct contact coculture system. (c) The knockdown efficiency of PI3K in chondrocytes was confirmed by qPCR and Western blotting. Values are expressed as mean ± s.d., ^∗∗∗^*p* < 0.001, ns indicates no significance. (d) Immunoblot images showing the effect of Kindlin-2 overexpression on the expression of p-PI3K/PI3K, p-AKT/AKT, and p-mTOR/mTOR in PI3K knockdown chondrocytes in the direct contact coculture system.
