# Supplementary material for: Kindlin-2 Promotes Chondrogenesis and Ameliorates IL-1beta-Induced Inflammation in Chondrocytes Cocultured with BMSCs in the Direct Contact Coculture System
Source: Oxid Med Cell Longev. 2022 Apr 12;2022:3156245. doi: 10.1155/2022/3156245 (PMC9018182; doi:10.1155/2022/3156245)
Supplement: Supplementary Materials — Supplementary Figure 1: Kindlin-2 mediates PI3K/AKT/mTOR signaling pathway in chondrocytes. (A) Overexpression efficiency of PI3K in chondrocytes was confirmed by qPCR and Western blotting. Values are expressed as mean ± s.d., ∗∗∗p < 0.001. (b) Immunoblot images showing the effect of Kindlin-2 knockdown on the expression of p-PI3K/PI3K, p-AKT/AKT, and p-mTOR/mTOR in PI3K overexpressed chondrocytes in the direct contact coculture system. (c) The knockdown efficiency of PI3K in chondrocytes was confirmed by qPCR and Western blotting. Values are expressed as mean ± s.d., ∗∗∗p < 0.001, ns indicates no significance. (d) Immunoblot images showing the effect of Kindlin-2 overexpression on the expression of p-PI3K/PI3K, p-AKT/AKT, and p-mTOR/mTOR in PI3K knockdown chondrocytes in the direct contact coculture system. [file 3156245.f1.docx]

**Kindlin-2 promotes chondrogenesis and ameliorates IL-1beta-induced inflammation in chondrocytes cocultured with BMSCs** **in the direct contact coculture system**

Zhefeng Chen^1,^^*^, Kai Shen^1,*^, Ziyang Zheng^1,*^, Jinchun Zhou^1,*^, Shujie Zhao^1^, Huanghe Song^1^, Jiuxiang Liu^1^, Xuan Zhao^1^, Feng Liu^1,#^, Qiang Zuo^1,#^

^1^ Department of Orthopedics, The First Affiliated Hospital of Nanjing Medical University, Nanjing, 210029, China.

^#^Corresponding author.

Feng Liu: njliuf@hotmail.com; Qiang Zuo: zuoqiang1985@njmu.edu.cn

^*^These authors contributed equally to this work.

**Supplementary material**


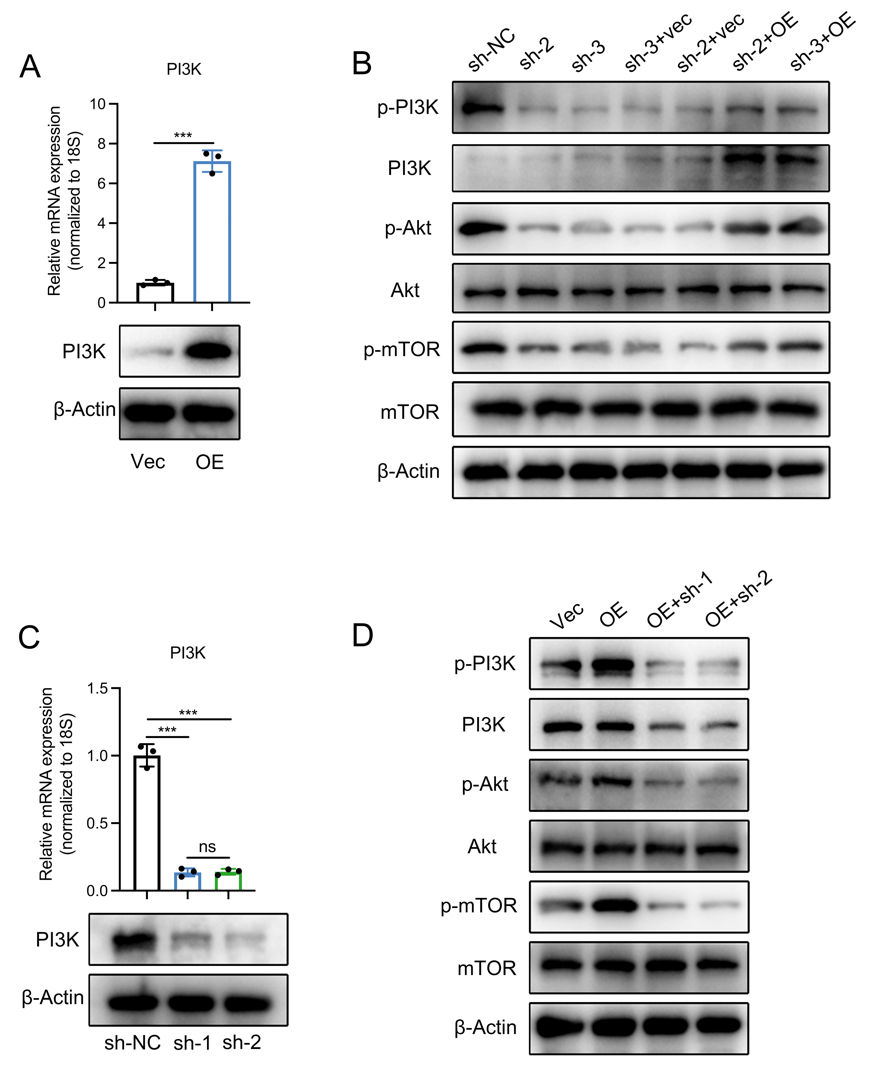


**Supplementary Figure 1. Kindlin-2 mediates PI3K/AKT/mTOR signaling pathway in chondrocytes.**

(A) Overexpression efficiency of PI3K in chondrocytes was confirmed by qPCR and Western blotting. Values are expressed as mean ± s.d., ***p < 0.001.

(B) Immunoblot images showing the effect of Kindlin-2 knockdown on the expression of p-PI3K/PI3K, p-AKT/AKT, and p-mTOR/mTOR in PI3K overexpressed chondrocytes in the direct contact coculture system.

(C) The knockdown efficiency of PI3K in chondrocytes was confirmed by qPCR and Western blotting. Values are expressed as mean ± s.d., ***p < 0.001, ns indicates no significance.

(D) Immunoblot images showing the effect of Kindlin-2 overexpression on the expression of p-PI3K/PI3K, p-AKT/AKT, and p-mTOR/mTOR in PI3K knockdown chondrocytes in the direct contact coculture system.
